# Cabin temperature during prehospital patient transport – a prospective observational study

**DOI:** 10.1186/s13049-020-00759-0

**Published:** 2020-07-13

**Authors:** Tuva Svendsen, Inger Lund-Kordahl, Knut Fredriksen

**Affiliations:** 1 Anaesthesia and Critical Care Research Group, Faculty of Healthcare Sciences, UiT-the Arctic University of Norway, Tromsø, Norway; 2 Division of Emergency Medical Services, University Hospital of North Norway, Tromsø, Norway

**Keywords:** Admission hypothermia, Ambient temperature, Cold exposure, Pre-hospital transport, HEMS, Helicopter emergency medical service, Ambulance

## Abstract

**Background:**

Few studies have investigated the patient compartment temperatures during ambulance missions or its relation to admission hypothermia. Still hypothermia is a known risk factor for increased mortality and morbidity in both trauma and disease. This has special relevance to our sub-arctic region’s pre-hospital services, and we prospectively studied the environmental temperature in the patient transport compartment in both ground and air ambulances.

**Methods:**

We recorded cabin temperature during patient transport in two ground ambulances and one ambulance helicopter in the catchment area of the University Hospital of North Norway using automatic temperature loggers. The data were collected for one month in each of the four seasons. We calculated the sum of degrees Celsius below 18 min by minute to describe the patient exposure to unfavourably low cabin temperature, and present the data as box plots. The statistical differences between transport mode and season were analysed with ANCOVA.

**Results:**

The recorded cabin temperatures were higher during the summer than the other three seasons. However, we also found that helicopter transports were performed at lower cabin temperatures and with significantly more exposure to unfavourably low temperatures than the ground ambulance transports. Furthermore, the helicopter cabin reached the final temperature much slower than the ground ambulance cabins did or remained at a lower than comfortable temperature.

**Conclusions:**

Helicopter cabin temperature during ambulance missions should be monitored closer, particularly for patients at risk for developing admission hypothermia.

## Background

Tight thermoregulation is central to maintain normal human organ homeostasis. Thus, protecting patients from exposure to cold is important during pre-hospital care [1]. Even mild hypothermia is an additional physiologic burden for the acutely ill or injured patient [2]. This has particular relevance for the long-distance prehospital transports in the Northern regions of Scandinavia, however the incidence of admission hypothermia may be essentially the same for severely injured or diseased patients in different world regions, and it seems to be a constant threat during all seasons [1, 3–7].

Much of our knowledge about the relationship between core body temperature and patient outcome stems from research on perioperative patients [8]. During major surgery and operations lasting more than 30 min, the patient is at risk for becoming hypothermic. Anaesthesia contributes significantly to this temperature loss by inhibiting normal thermoregulatory mechanisms as vasoconstriction and shivering. Hence, the un-warmed patient typically loses 1–2 °C, and per-operative hypothermia increases the prevalence of coagulopathy, transfusion requirements, leads to more wound infections, delayed drug metabolism and prolonged recovery. In addition, the patients self-reported thermal discomfort was the most disturbing experience during the perioperative period [8].

Research on hypothermia in the pre-hospital setting has mainly focused on trauma patients. Most studies report an incidence below 10% in injured patients, but this depends on study population, method for temperature measurement, and cut off temperature for defining of hypothermia [1, 9–12]. Mild or moderate hypothermia is related to injury severity and may include every third patient. This means that the vulnerability for developing hypothermia differs among trauma patients, and the unconscious and most severely injured patients are at highest risk. Furthermore, hypothermia is associated with significantly increased mortality and morbidity [13–15]. Trauma patients in general anaesthesia are particularly prone to develop admission hypothermia and need active heating to preserve temperature [4].

The high prevalence of thermal discomfort during transport is particularly disturbing for the patients according to studies from Northern Sweden [16, 17]. Other Scandinavian publications have documented that simple equipment for patient insulation is carried by most ambulances, but active warming devices and even suitable thermometers to monitor patient temperature were not available in most vehicles [18, 19].

Even though that transport environment may be particularly important for patient temperature, few studies have directly addressed patient compartment temperature. Lapostolle and colleagues found that mobile unit temperature was an independent risk factor for development of hypothermia in trauma patients [13]. In an Australian helicopter emergency medical service (HEMS) study, no correlation was found between patient temperature and outdoor or cabin temperature. In this study, the best predictor for patient temperature after the transport was the patient’s temperature at the start of the mission, not the ambient temperature. However, none of the patients in this study that had been transported with a cabin temperature in the thermoneutral zone (TNZ) developed hypothermia, and the numbers were rather small [20]. For this reason, we believe that the relationship between ambient temperature and patient risks is still not firmly established. Knowledge about ambient temperature during prehospital transport is a prerequisite to offer optimal temperature conditions and to avoid further loss of patient body temperature. We believe that establishing firm knowledge about this is necessary to enable patient-directed quality changes in temperature control.

Because of the apparent lack of studies on ambient temperature conditions during patient transport, and the clear relevance this has to prehospital care in our subarctic setting, we have prospectively investigated the cabin temperature in ground and air ambulances during patient transports at different outdoor temperatures.

## Methods

### Study design

The study was a prospective observational study of prehospital patient compartment temperature in ground ambulances and HEMS in the catchment area of the University Hospital of North Norway (UNN) in Tromsø (UNN Tromsø).

### Setting

The UNN hospitals serve a widespread mixed urban/rural population. Approximately 60% of the population lives in urban areas, with short distance to one of the UNN hospitals. The rest live in rural areas, with up to 4 h transport time to hospital with ground ambulance. With one of the two HEMS in the area, the transport time is considerably shorter, with an average of ca 20 min, and a maximum of 50 min.

We included two ground ambulance stations from the UNN Ambulance Service, one in an inland municipality, operating mainly in a continental climate (Andslimoen, 120 min from UNN Tromsø, referred to as GA1), and the other in a coastal climate (Vollan, located 55 min from UNN Tromsø, referred to as GA2). In addition, we included the HEMS based at the UNN Tromsø.

The climate of the UNN catchment area is boreal, and differs between inland areas with low winter temperatures and warm summers, and a coastal zone with milder winters and summers. GA2 operates mainly in the coastal climate, and the GA1 always starts out in a continental climate, and sometimes the missions end in the coastal climate in the city of Tromsø. The HEMS is located in the city of Tromsø and serves both coastal and continental regions in its catchment area. The HEMS aircraft is a large Leonardo AW 139 with a spacious cabin that may transport one or two patients on stretcher.

### Patient compartment temperature

Cabin temperature was recorded automatically every minute with an Elpro Libero Ti1 temperature logger (Elpro-Buchs AG, Buchs, Switzerland). This logger is calibrated by an accredited laboratory and has an accuracy of ±0.2 °C in the range − 10 °C to + 25 °C. Two temperature loggers were placed in each included ambulance unit. One on the rear cabin wall, close to the cabin door, and within 30 cm from the patient head in the middle of the cabin. Both loggers were openly exposed to cabin air, well away from heater outlets. Temperatures were sampled for 30 day periods through November 2016, and February, May and July/August 2017. The recorded data were transferred from the logger to an Excel 2016 worksheet (Microsoft) with elproLOG ANALYZE software (V. 3.62.03, Elpro-Buchs, Switzerland). We used the mean temperature between the two loggers for calculations when the temperatures differed.

### Time points for patient transports

Time points for start and stop for all actual patient transports during the four study periods were recorded from the ambulance records, and air ambulance database.

### Defining the exposure to unfavourably low temperature - “cold exposure”

We chose 18 °C as a “neutral” cabin temperature, because all ambulance units in the healthcare trust have duvets to cover the patients with during transport, and most patients are normally fully dressed during the transport. Patient exposure for low temperature during transport was calculated as the sum of numerical deviations from 18 °C for the first 5, 10 or 15 min of the transport. We chose a maximum of 15 min in order to avoid the effect of longer transport times for the ground ambulances. Hereafter, we refer to this sum of deviations as the “cold exposure”.

### Statistical analysis

In order to analyse the difference between the three ambulance units on cold exposure, controlling for season, we conducted a One-way ANCOVA using IBM SPSS Statistics version 16.0 (IBM Corp., Armonk, New York). When the ANCOVA yielded a significant variation, we calculated a Tukey post hoc test (Tukey’s Honest Significant Difference test) for between-group comparisons and considered *p* ≤ 0.05 as significant.

## Results

Table 1 presents the 453 ground ambulance- and HEMS-missions included in the study, divided into ambulance vehicles and season.

**Table 1:** Air and ground ambulance missions

	GA1 (Andslimoen)	GA2 (Vollan)	HEMS(Tromsø)	Total
November	27 (6,0%)	27 (6,0%)	33 (7,3%)	87 (19,2%)
February	33 (7,3%)	38 (8,4%)	33 (7,3%)	104 (23,0%)
May	41 (9,0%)	40 (8,8%)	56 (12,4%)	137 (30,2%)
July/August	36 (7,9%)	33 (7,3%)	56 (12,4%)	125 (27,6%)
Total	137 (30,2%)	138 (30,5%)	178 (39,3%)	453 (100%)

Number and percent of air and ambulance missions divided into seasons and locations. HEMS: helicopter emergency medical service. *GA* ground ambulance

Before further calculations, we explored the difference between temperatures recorded simultaneously by the pair of loggers (data not shown). The highest discrepancy between temperatures was found in the ground ambulances (range 1.0–5.0). The lowest temperature was always recorded by the logger close to the cabin door. The corresponding difference for the HEMS was clearly smaller (range 0.2–1.0 °C). We used the mean between the two observations in the rest of the study when they differed.

**Fig. 1:**
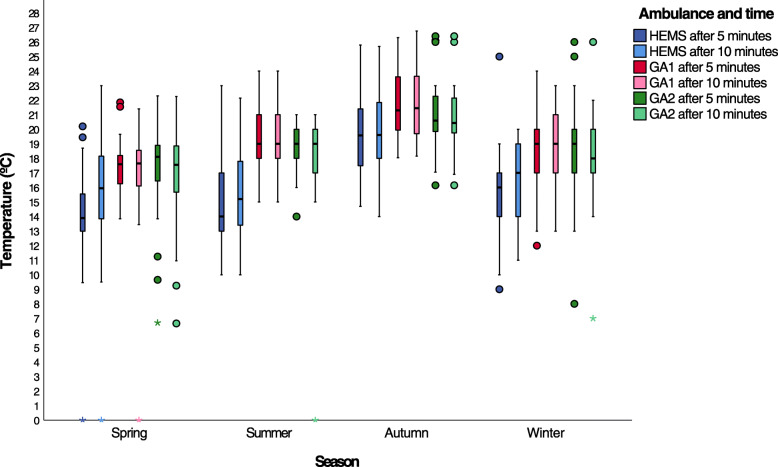
Ambulance cabin temperature after 5 and 10 min. Patient compartment temperatures for 453 missions 5 min and 10 min after patient transport started shown as box plots for the three ground and air ambulances and four seasons. HEMS: helicopter emergency medical service, GA1 and GA2: ground ambulance 1 and 2

To reduce the effect of the cold air from opening cabin doors during patient loading, we present the temperatures recorded five and ten minutes into the missions. Figure 1 shows the temperature means and distribution after five and ten minutes as box plots. The HEMS cabin mean temperature varied between 9 and 25 °C after five minutes, and the recordings in the two ground ambulance varied between 12 and 24 °C, and 13–22 °C, respectively. This trend was essentially the same after 10 min, and HEMS temperatures were lower than for the ground ambulances. Summer temperatures were as expected higher than for the other seasons, and it seemed that this was most prominent for the HEMS cabin. The boxplots also suggest that the increase in cabin temperature from five to ten minutes was larger in the HEMS than the ground ambulances.

**Fig. 2:**
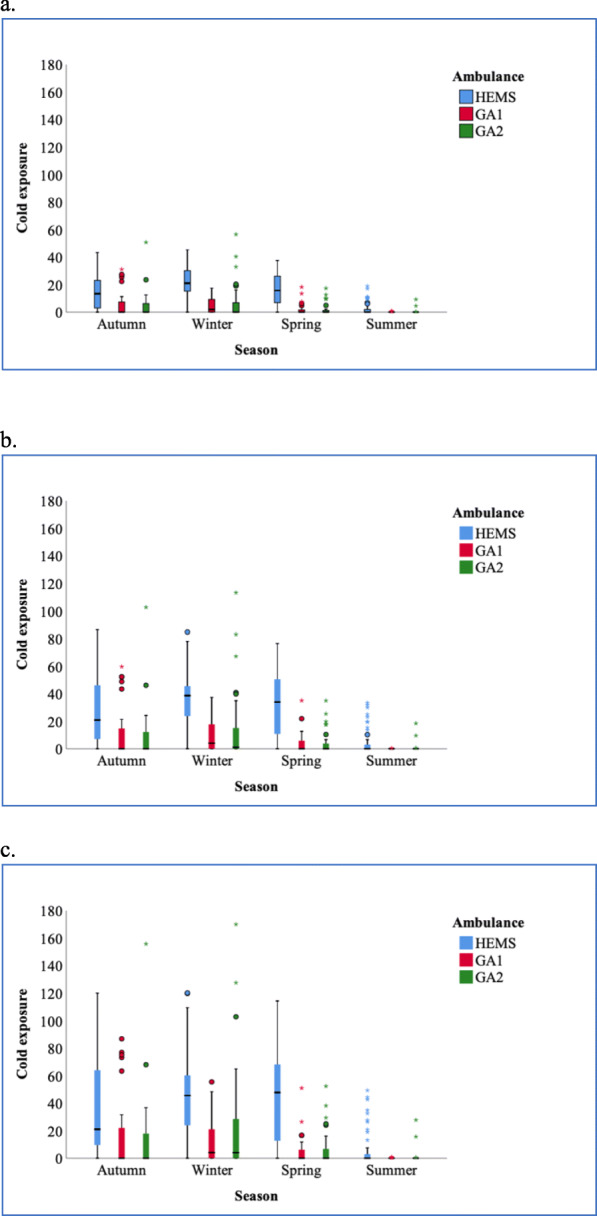
Cold exposure in ground and HEMS missions. Patient exposure for unfavourably low temperature during transport was calculated as the sum of numerical deviations from a chosen “neutral” temperature of 18 °C, minute by minute, in 5 (**a**), 10 (**b**) and 15 min (**c**) after transport started. This calculated figure was termed “cold exposure”. The results are shown as box plots for the three ground and air ambulances and four seasons. The boxes contain the inter-quartile difference with the median. HEMS: helicopter emergency medical service, GA1 and GA2: ground ambulance 1 and 2

To compare statistically the differences in exposure to unfavourably low temperatures between vehicles and seasons, we calculated the *cold exposure*, defined by us as the sum of degrees below 18 °C minute by minute during the first 5, 10 and 15 min. We present the results as box plots in Fig. 2. In order to distinguish between the co-existing effects of season and mode of transport, we analysed cold exposure statistically. We found a significant effect between HEMS and the two ground ambulances after controlling for season at 15 min in an ANCOVA (F (2,419) = 42,490, *p* < 0,05). We also found a significant effect of summer season vs the three other seasons on cold exposure after controlling for vehicle (F (3,418) = 22,802, *p* < 0,05).

**Fig. 3:**
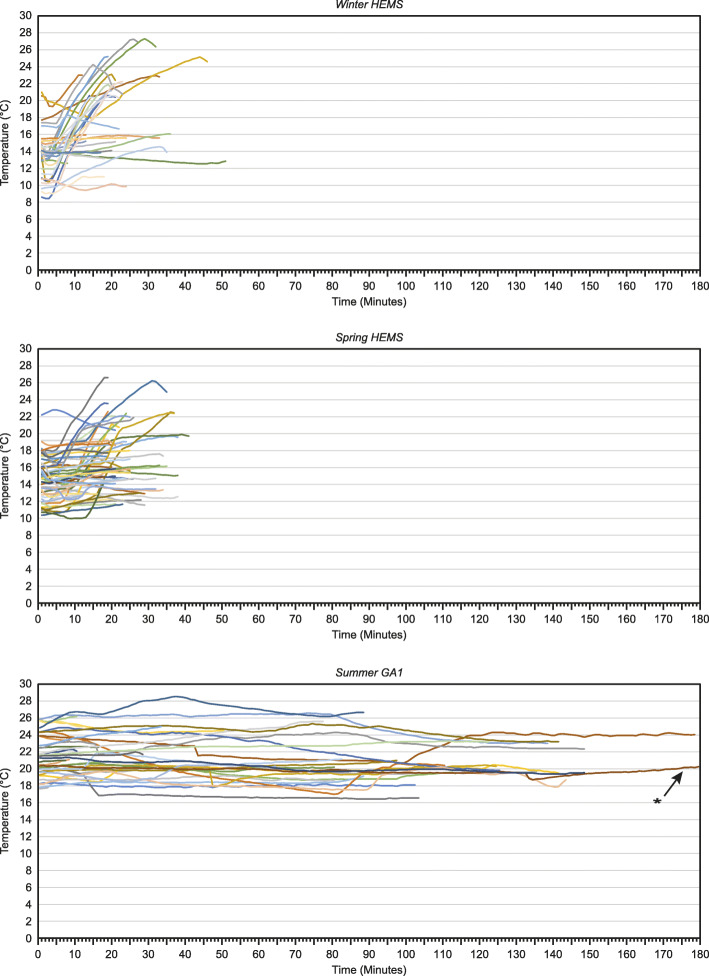
Minute by minute temperature recordings during patient transport. Patient compartment temperatures, minute by minute, in the HEMS during winter (**a**), and spring (**b**), and in one ground ambulance during summer (**c**). The three graphs are selected to illustrate the main temperature trends between different vehicles and seasons. The complete data for all three ambulance vehicles and four seasons is submitted as Additional data file. The asterisk denotes that the graph was truncated at 180 min for this individual mission. The temperature remained essentially unchanged until the end of the mission at 256 min. HEMS: helicopter emergency medical service

In Additional file 1, we present 12 graphs that show how the cabin temperature develops minute by minute from start to end of all 453 patient transports for all four seasons and three ambulances. Figure 3 shows a selection of three graphs that illustrate the main findings for all ambulances and seasons. Notably, the HEMS has much shorter transport times than ground ambulances, but it takes longer to reach the final temperature, compared with the ground ambulances.

## Discussion

In the present study, we used simple temperature loggers to record the temperature in the patient compartments in three ground and air ambulance units representative for pre-hospital transport in Northern Norway. As expected, we found that cabin temperatures were significantly higher during the summer month compared to the other seasons. However, it was a less expected finding that the HEMS unit generally transported patients at lower cabin temperature than the ground units did. This is alarming, because HEMS transports patients with a higher average severity of disease or trauma than the ground ambulances do, and are thus more vulnerable to develop admission hypothermia. Furthermore, the temperature development plots demonstrate that whereas ground ambulances reach the final temperature plateau within few minutes, and keep the temperature at this level throughout the transport time, HEMS uses a significant part of the flight time to reach this temperature. HEMS temperature plots even show that temperatures may remain stable between 10 and 18 °C during the entire flight.

Since we terminated our calculations at 15 min into the missions, the results we report are most probably conservative estimates. The maximum temperature had not been reached at 15 min in some of the HEMS missions. This means that the difference between ground and air ambulances was theoretically even larger than reported here.

Most ambulance patients are dressed when transported, and both air and ground ambulances in this region regularly uses a duvet instead of a thin blanket during the transport. Therefore, the low temperatures reported in this study may not be experienced as uncomfortable by all patients. In ground ambulances patients may also easily communicate with the personnel and ask for more heat or the duvet if they feel cold. However, this is not the case in the HEMS. Communication with the crew is difficult because of the noise in the helicopter cabin. At least to some degree, this problem may be overcome if the patient is equipped with a headset with earphones and microphone, but this is not regularly used. In addition, it is less convenient to change the bedding during flight, since both patient and the crew must keep the safety harness on during the entire flight. We believe that it will not have as high priority as other necessary in-flight work during the short transport time of a typical HEMS mission, and we believe that HEMS patients because of all these reasons are at bigger risk for becoming hypothermic than ground ambulance patients.

In addition, whereas ground ambulances have a thermostat driven heating system that can be regulated by the cabin personnel, helicopter heating must be completely off during take-off and landing due to engine performance issues and the heating may be regulated by the cockpit crew members only. We assume that this also contributes to a less active use of the heating during flight. Heating is also not possible when the aircraft is on ground, irrespective of whether the engine is turned off or on idle. The cabin doors used for loading and unloading the patient are wide sliding doors that virtually expose the entire cabin interior to outside temperature when open. For this reason, the cabin temperature will normally be close to the outdoor temperature at the start of the flight.

Another important difference between HEMS and ground ambulances is that flying personnel in the North Norwegian services regularly are dressed to meet the outdoor climate conditions at an austere landing site during the entire mission, and it is not feasible to change clothing when first seated, at least not for cockpit crew. It is even common for flying personnel to wear a survival (immersion) suit long periods of the year. It is therefore tempting to assume that in this situation, regulating the cabin heating will be guided primarily by the thermal comfort of the crew and not the needs of the patient. However, this assumption has not been addressed here, but clearly represents a topic that should be followed up in a future study.

During the study period, all ambulance and HEMS crew were aware of that temperature loggers were placed in the patient compartments as part of a research project. However, we have assumed that this did not change the personnel’s heating policy during the study period. The personnel had received a short informative notice about the project, but no information that could raise the assumption that the research team suspected suboptimal temperatures during patient transport. We would especially not expect that it led to different behaviour in ground ambulances and HEMS, in favour of more active temperature control in the ground ambulance units. For this reason, we assume that the recordings represent the typical cabin temperature in our services.

An important unresolved question for the present study is what temperature is desirable in an ambulance patient compartment. The current literature does not answer this question, but much research on the TNZ (the temperature range where body temperature is maintained without need for changes in normal metabolic rate) of humans suggests that a temperature of 18–22 °C will be comfortable for a dressed person [21, 22]. However, the lower critical temperature is affected by a number of factors, like body mass index, age and disease, and of course the amount of clothing and additional insulation. Thus, the TNZ term is difficult to apply in the setting of the present study. As mentioned earlier, much of the existing research in this field is from the operating rooms, and here, the core temperature of the undressed and exposed patient is monitored closely. Even under these controlled circumstances, TNZ may vary and most patients will need active external warming to conserve body temperature during the anaesthesia and operation [8]. For this reason, we had to choose a pragmatic lower temperature that we considered “safe”, and we assumed that an ambient temperature around 18–22 °C would be acceptable for most ambulance patients. The assumption was simply based on what is considered comfortable normal room temperature in Scandinavian houses. By use of a blanket or duvet, the patient environment temperature can easily be regulated if the patient compartment is close to this temperature.

Obviously, patient ability to self-regulate their temperature differs much, and a number of medical conditions, major trauma and general anaesthesia make selected patients particularly prone to develop admission hypothermia [1, 4, 23]. The most vulnerable patients may in addition be repeatedly uncovered because of medical procedures. The need for more intensive electro-medical monitoring leads to more cables that may have to be accessed inflight, and there is more active use of vascular lines and other equipment that leads to uncovering the patient. The UNN HEMS aims at short on ground times to reach the receiving hospital fast. Therefore, some procedures like cannulation and especially ultrasound examination are often performed *en route* to hospital. Repeated uncovering is thus particularly common in the patients that already are at higher risk of becoming hypothermic. This fact, together with the results of the present study, supports the notion that pre-hospital personnel should aim for a stricter control of cabin temperature in vulnerable patients.

In an attempt to describe deviation from desired temperature, we coined the term “cold exposure”, which is the sum of degrees below 18 °C for a defined time after the start of patient transport. Even though the lower limit for an assumed favourable temperature was pragmatically chosen as 18 °C it enabled us to describe numerically the combined effect of magnitude of the cold and the length of exposure. Thus, we could compare and analyse statistically differences between the patient compartment temperatures in the vehicles. We conclude from our results that the temperature issue is a particularly important problem in the HEMS setting, even though HEMS missions have shorter duration than ground ambulance missions have in our rural areas. The difference between HEMS and ground ambulance is significant, even when correcting for the effect of season. The results also support the notion that there is a difference between seasons, as cold exposure during summer was significantly lower than the other three seasons, at least in our setting. Some other studies have arrived at the same conclusion, whereas other have not [3–7, 20]. However, the studies may not be comparable, as they were performed in different regions of the world, with different winter temperatures.

One limitation of our study is that the cabin air temperature is not directly related to neither the patient’s risk for developing hypothermia nor thermal comfort. However, we believe that it is important for regulating the patient environment, especially for vulnerable subgroups that may not be able to influence their thermal comfort. The fact that we included all patients transported during the study period, and did not correct for individual clothing, duvet cover, or other individual factors also limits what can be concluded from our results. Still, we believe that firm evidence about the ambient temperature is important to improve conservation of patient temperature in pre-hospital care. We have also noted several points of interest for future research, like the impact of personnel thermal comfort, and personnel knowledge and attitudes to temperature monitoring and cabin temperature. Much is also still unknown about the seemingly complex relationship between the temperature that is unfavourable for the patient’s core temperature and the environmental temperature.

However, the results reported here support more active patient temperature control in the patient groups that are of highest risk for becoming cold. This is important for any pre-hospital care provider, but may obviously be an important issue to be addressed by future HEMS aircraft designers.

## Conclusions

During patient transport, the patient compartment temperatures in the HEMS were lower than in ground ambulances during all seasons. The amount of exposure to unfavourably low temperature was also significantly higher during HEMS transport. We discuss the reasons for this, and we suggest more focus on helicopter cabin temperatures during ambulance missions, at least for particularly the patients with high risk for developing admission hypothermia.

## Supplementary information

**Additional file 1.** Additional material showing the temperature plots for all three ambulance vehicles and all four seasons, is available as Additional file 1. The figures are A: HEMS winter, B: HEMS spring, C: HEMS summer, D: HEMS: autumn, E: GA1 winter, F: GA1 spring, G: GA1 summer, H: GA1 autumn, I: GA2 winter, J: GA2 spring, K: GA2 summer, L: GA2 autumn. The asterisk in Fig. G denotes that the graph was truncated at 180 min. The temperature remained essentially unchanged until the end of the mission at 256 min. HEMS: helicopter emergency medical service. GA: ground ambulance.

## Data Availability

The data are part of a quality registry at the UNN and thus not available for external users due to Health Trust data protection issues.

## Abbreviations

ANCOVA: Analysis of covariance
HEMS: Helicopter Emergency Medical Service
TNZ: Thermoneutral zone
UNN: University Hospital of North Norway
