# Supplementary figures and images for: Cabin temperature during prehospital patient transport – a prospective observational study
Source: Scand J Trauma Resusc Emerg Med. 2020 Jul 13;28:64. doi: 10.1186/s13049-020-00759-0 (PMC7359238; doi:10.1186/s13049-020-00759-0)

J

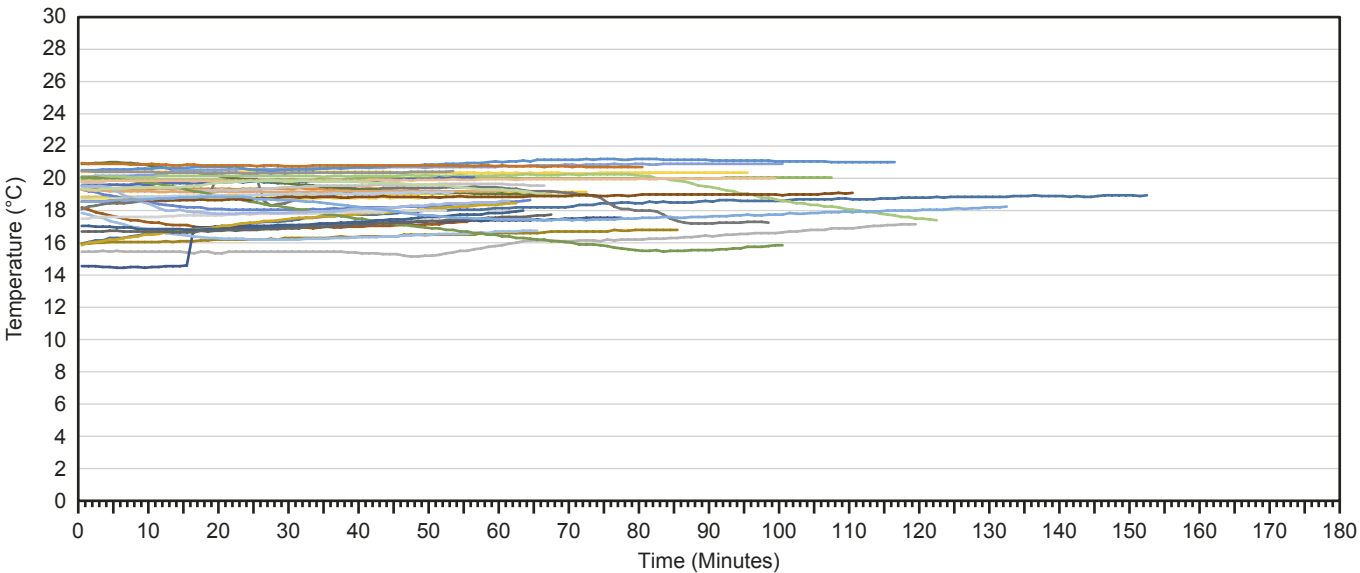

Supplement: Supplementary file 1 — Additional file 1. Additional material showing the temperature plots for all three ambulance vehicles and all four seasons, is available as Additional file 1. The figures are A: HEMS winter, B: HEMS spring, C: HEMS summer, D: HEMS: autumn, E: GA1 winter, F: GA1 spring, G: GA1 summer, H: GA1 autumn, I: GA2 winter, J: GA2 spring, K: GA2 summer, L: GA2 autumn. The asterisk in Fig. G denotes that the graph was truncated at 180 min. The temperature remained essentially unchanged until the end of the mission at 256 min. HEMS: helicopter emergency medical service. GA: ground ambulance. [file 13049_2020_759_MOESM1_ESM.zip › 10-J v2.pdf]

**K**

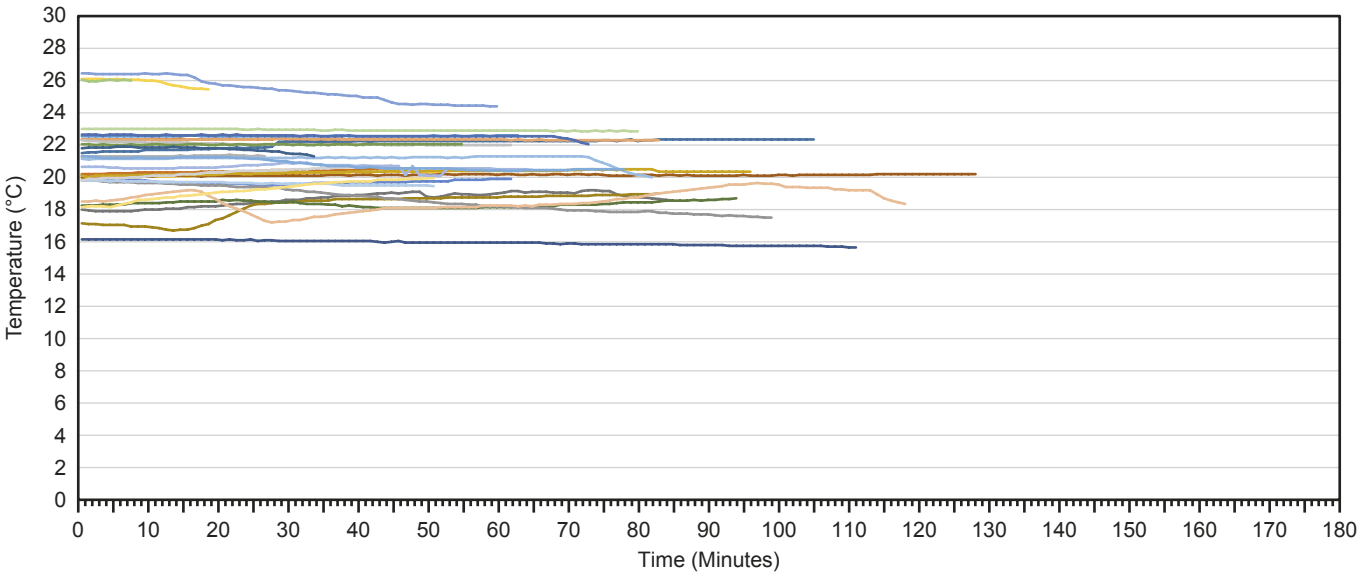

Supplement: Supplementary file 1 — Additional file 1. Additional material showing the temperature plots for all three ambulance vehicles and all four seasons, is available as Additional file 1. The figures are A: HEMS winter, B: HEMS spring, C: HEMS summer, D: HEMS: autumn, E: GA1 winter, F: GA1 spring, G: GA1 summer, H: GA1 autumn, I: GA2 winter, J: GA2 spring, K: GA2 summer, L: GA2 autumn. The asterisk in Fig. G denotes that the graph was truncated at 180 min. The temperature remained essentially unchanged until the end of the mission at 256 min. HEMS: helicopter emergency medical service. GA: ground ambulance. [file 13049_2020_759_MOESM1_ESM.zip › 11-K v2.pdf]

L

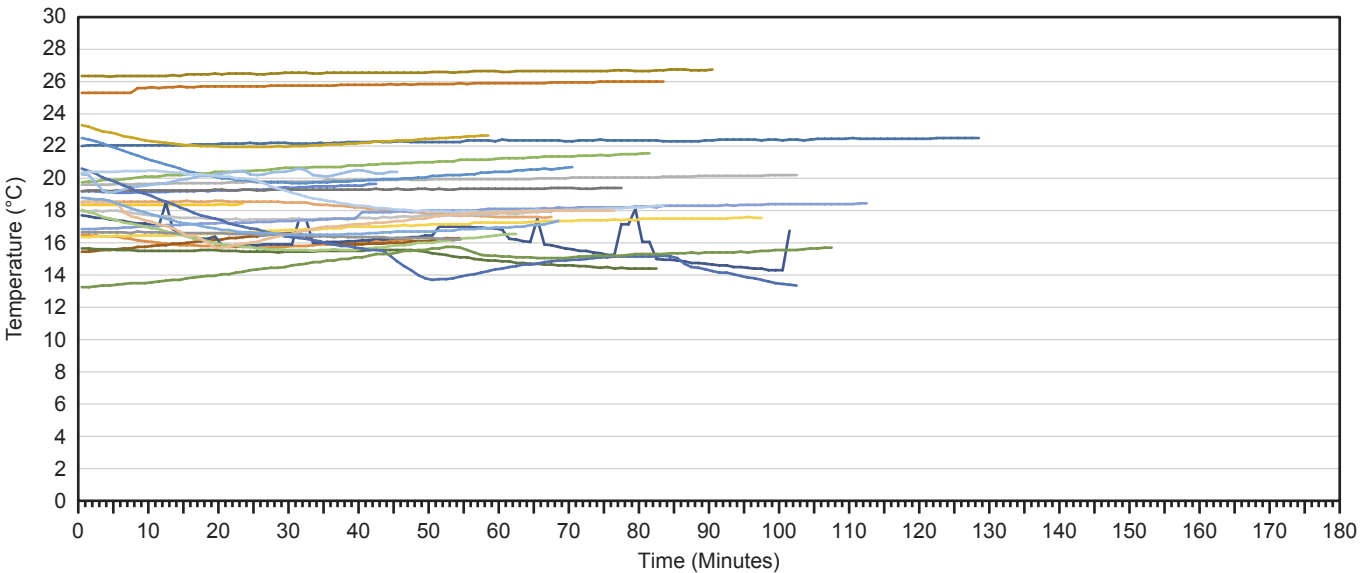

Supplement: Supplementary file 1 — Additional file 1. Additional material showing the temperature plots for all three ambulance vehicles and all four seasons, is available as Additional file 1. The figures are A: HEMS winter, B: HEMS spring, C: HEMS summer, D: HEMS: autumn, E: GA1 winter, F: GA1 spring, G: GA1 summer, H: GA1 autumn, I: GA2 winter, J: GA2 spring, K: GA2 summer, L: GA2 autumn. The asterisk in Fig. G denotes that the graph was truncated at 180 min. The temperature remained essentially unchanged until the end of the mission at 256 min. HEMS: helicopter emergency medical service. GA: ground ambulance. [file 13049_2020_759_MOESM1_ESM.zip › 12-L v2.pdf]

A

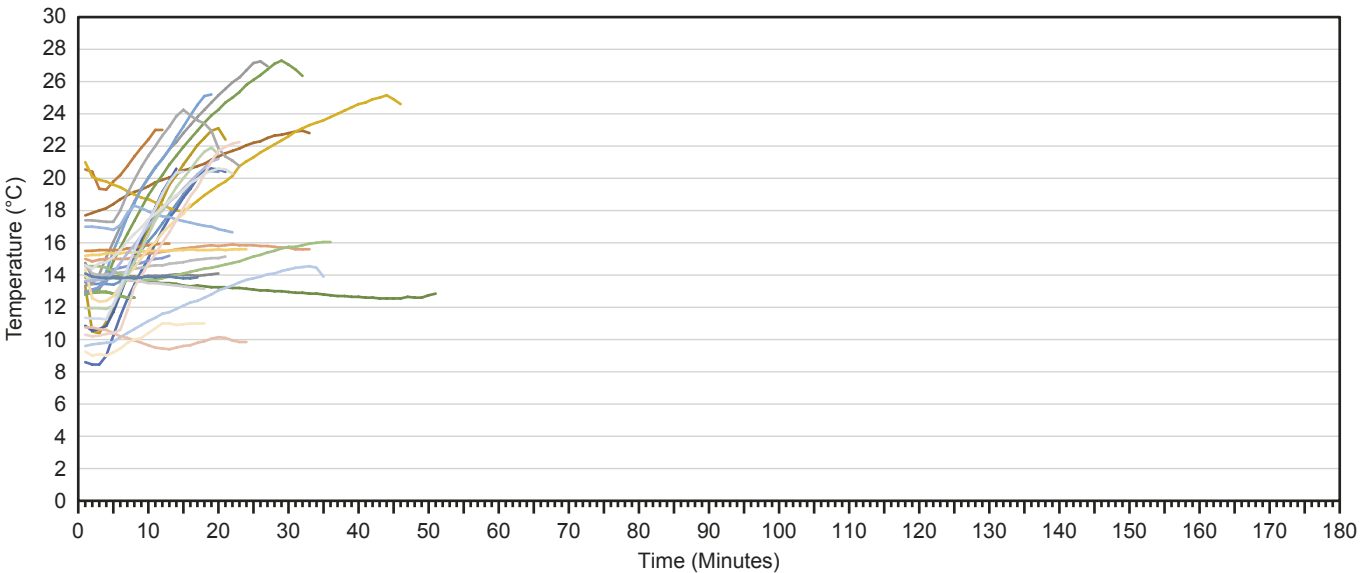

Supplement: Supplementary file 1 — Additional file 1. Additional material showing the temperature plots for all three ambulance vehicles and all four seasons, is available as Additional file 1. The figures are A: HEMS winter, B: HEMS spring, C: HEMS summer, D: HEMS: autumn, E: GA1 winter, F: GA1 spring, G: GA1 summer, H: GA1 autumn, I: GA2 winter, J: GA2 spring, K: GA2 summer, L: GA2 autumn. The asterisk in Fig. G denotes that the graph was truncated at 180 min. The temperature remained essentially unchanged until the end of the mission at 256 min. HEMS: helicopter emergency medical service. GA: ground ambulance. [file 13049_2020_759_MOESM1_ESM.zip › 1-A v2.pdf]

B

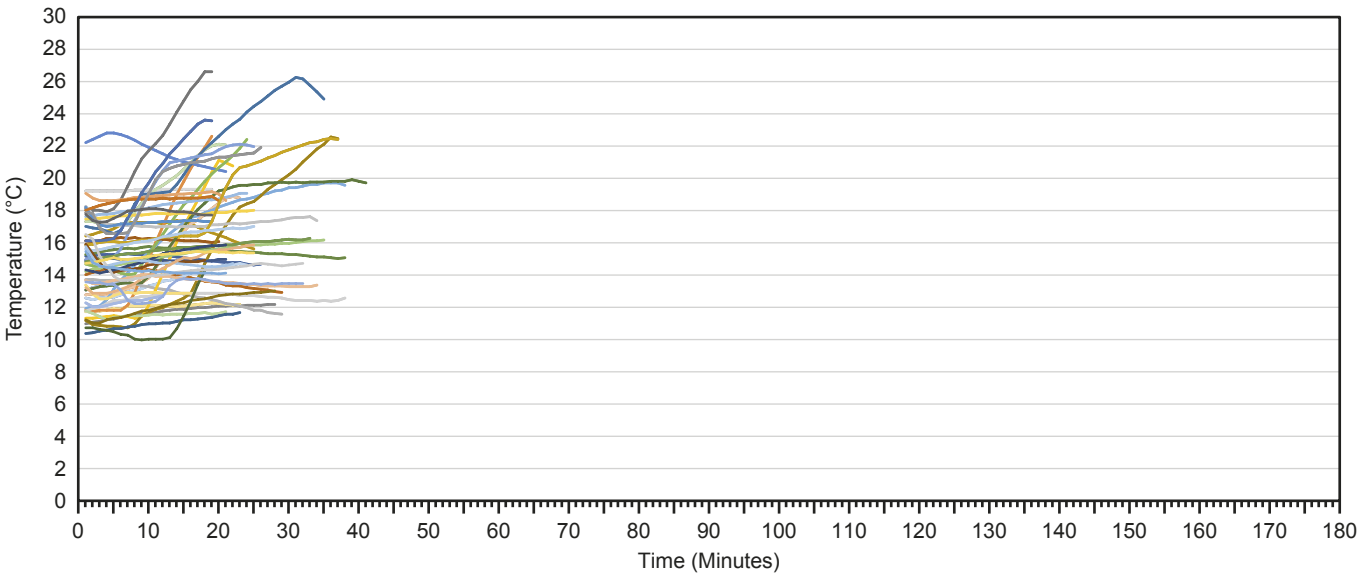

Supplement: Supplementary file 1 — Additional file 1. Additional material showing the temperature plots for all three ambulance vehicles and all four seasons, is available as Additional file 1. The figures are A: HEMS winter, B: HEMS spring, C: HEMS summer, D: HEMS: autumn, E: GA1 winter, F: GA1 spring, G: GA1 summer, H: GA1 autumn, I: GA2 winter, J: GA2 spring, K: GA2 summer, L: GA2 autumn. The asterisk in Fig. G denotes that the graph was truncated at 180 min. The temperature remained essentially unchanged until the end of the mission at 256 min. HEMS: helicopter emergency medical service. GA: ground ambulance. [file 13049_2020_759_MOESM1_ESM.zip › 2-B v2.pdf]

C

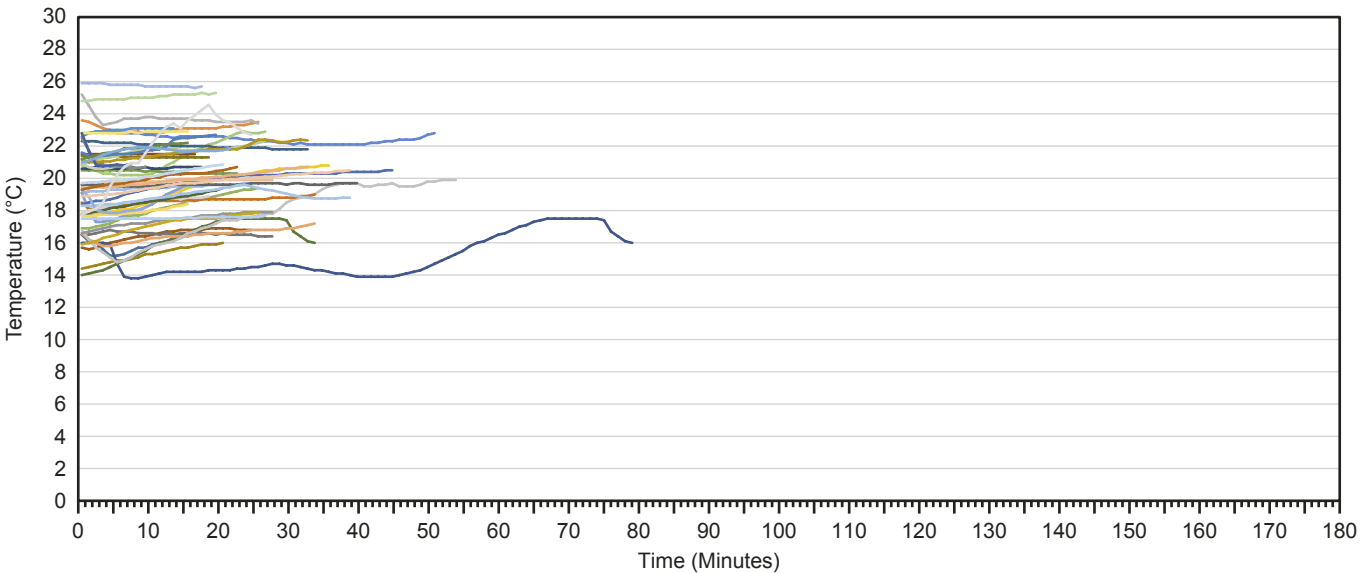

Supplement: Supplementary file 1 — Additional file 1. Additional material showing the temperature plots for all three ambulance vehicles and all four seasons, is available as Additional file 1. The figures are A: HEMS winter, B: HEMS spring, C: HEMS summer, D: HEMS: autumn, E: GA1 winter, F: GA1 spring, G: GA1 summer, H: GA1 autumn, I: GA2 winter, J: GA2 spring, K: GA2 summer, L: GA2 autumn. The asterisk in Fig. G denotes that the graph was truncated at 180 min. The temperature remained essentially unchanged until the end of the mission at 256 min. HEMS: helicopter emergency medical service. GA: ground ambulance. [file 13049_2020_759_MOESM1_ESM.zip › 3-C v2.pdf]

D

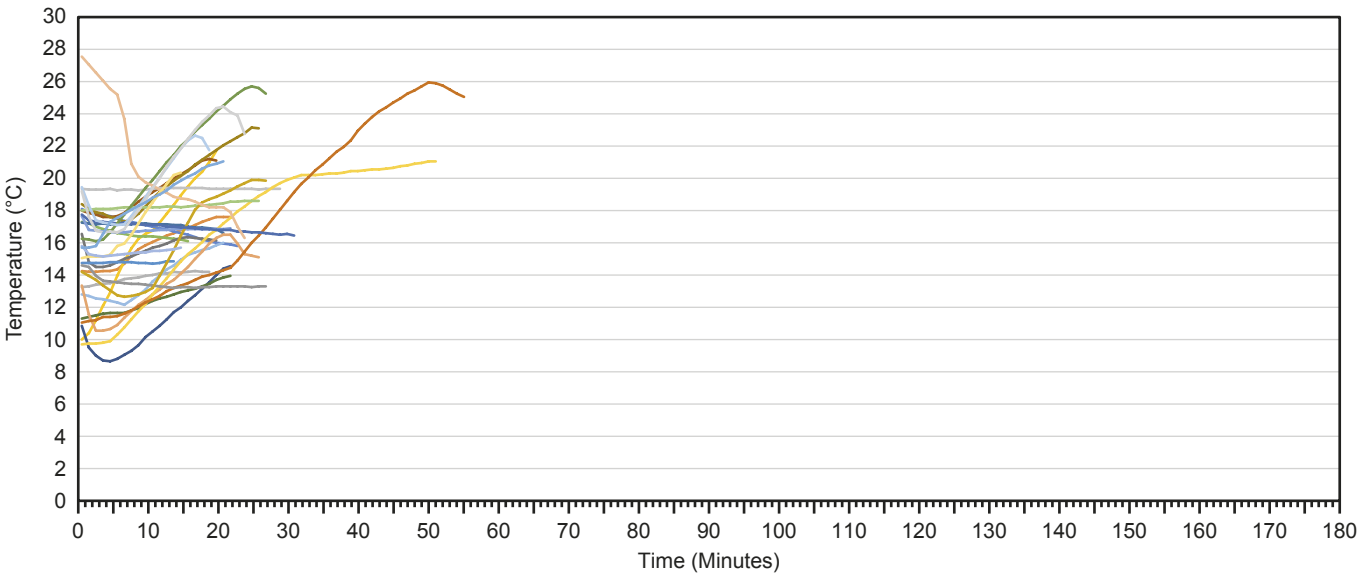

Supplement: Supplementary file 1 — Additional file 1. Additional material showing the temperature plots for all three ambulance vehicles and all four seasons, is available as Additional file 1. The figures are A: HEMS winter, B: HEMS spring, C: HEMS summer, D: HEMS: autumn, E: GA1 winter, F: GA1 spring, G: GA1 summer, H: GA1 autumn, I: GA2 winter, J: GA2 spring, K: GA2 summer, L: GA2 autumn. The asterisk in Fig. G denotes that the graph was truncated at 180 min. The temperature remained essentially unchanged until the end of the mission at 256 min. HEMS: helicopter emergency medical service. GA: ground ambulance. [file 13049_2020_759_MOESM1_ESM.zip › 4-D v2.pdf]

E

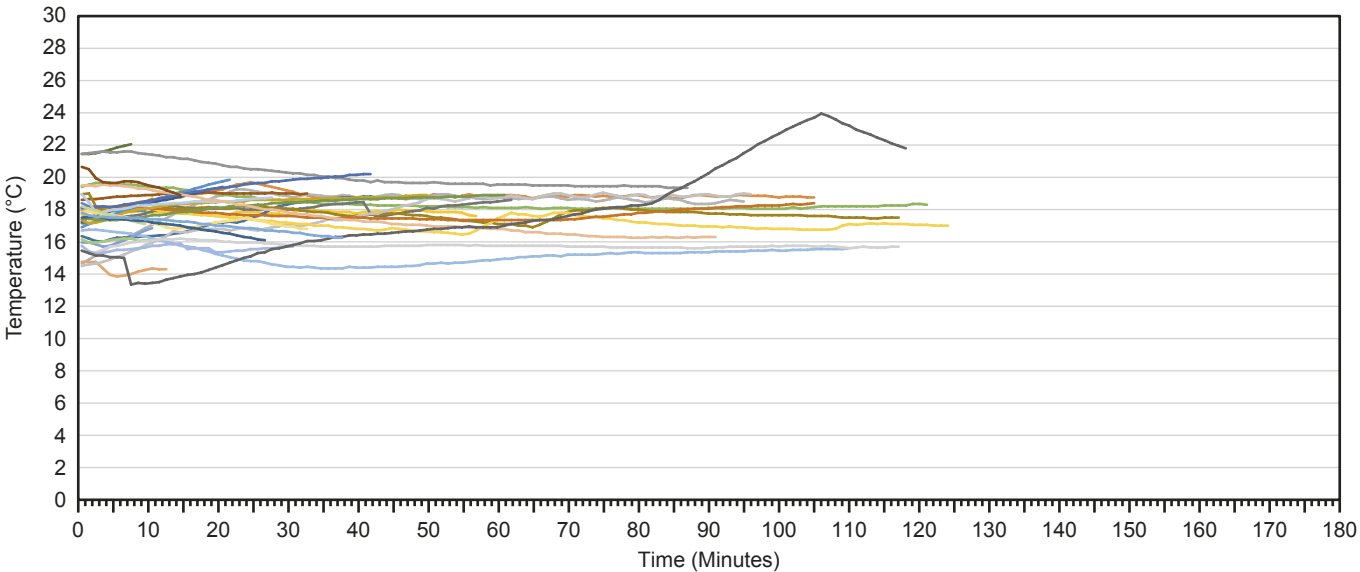

Supplement: Supplementary file 1 — Additional file 1. Additional material showing the temperature plots for all three ambulance vehicles and all four seasons, is available as Additional file 1. The figures are A: HEMS winter, B: HEMS spring, C: HEMS summer, D: HEMS: autumn, E: GA1 winter, F: GA1 spring, G: GA1 summer, H: GA1 autumn, I: GA2 winter, J: GA2 spring, K: GA2 summer, L: GA2 autumn. The asterisk in Fig. G denotes that the graph was truncated at 180 min. The temperature remained essentially unchanged until the end of the mission at 256 min. HEMS: helicopter emergency medical service. GA: ground ambulance. [file 13049_2020_759_MOESM1_ESM.zip › 5-E v2.pdf]

F

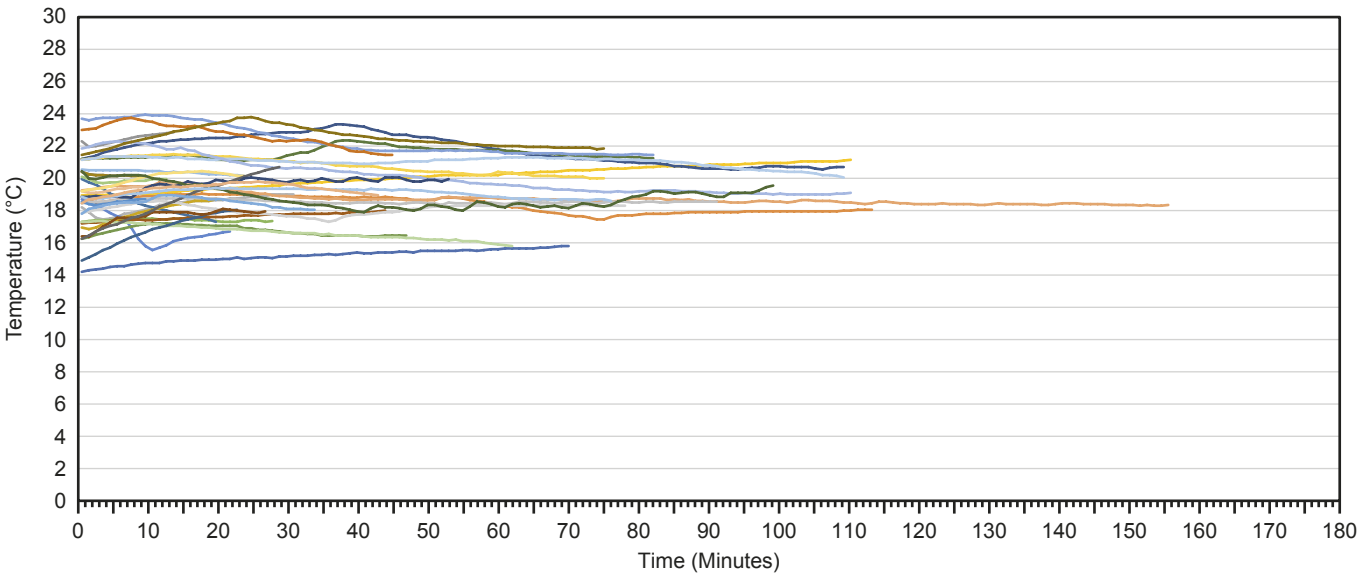

Supplement: Supplementary file 1 — Additional file 1. Additional material showing the temperature plots for all three ambulance vehicles and all four seasons, is available as Additional file 1. The figures are A: HEMS winter, B: HEMS spring, C: HEMS summer, D: HEMS: autumn, E: GA1 winter, F: GA1 spring, G: GA1 summer, H: GA1 autumn, I: GA2 winter, J: GA2 spring, K: GA2 summer, L: GA2 autumn. The asterisk in Fig. G denotes that the graph was truncated at 180 min. The temperature remained essentially unchanged until the end of the mission at 256 min. HEMS: helicopter emergency medical service. GA: ground ambulance. [file 13049_2020_759_MOESM1_ESM.zip › 6-F v2.pdf]

G

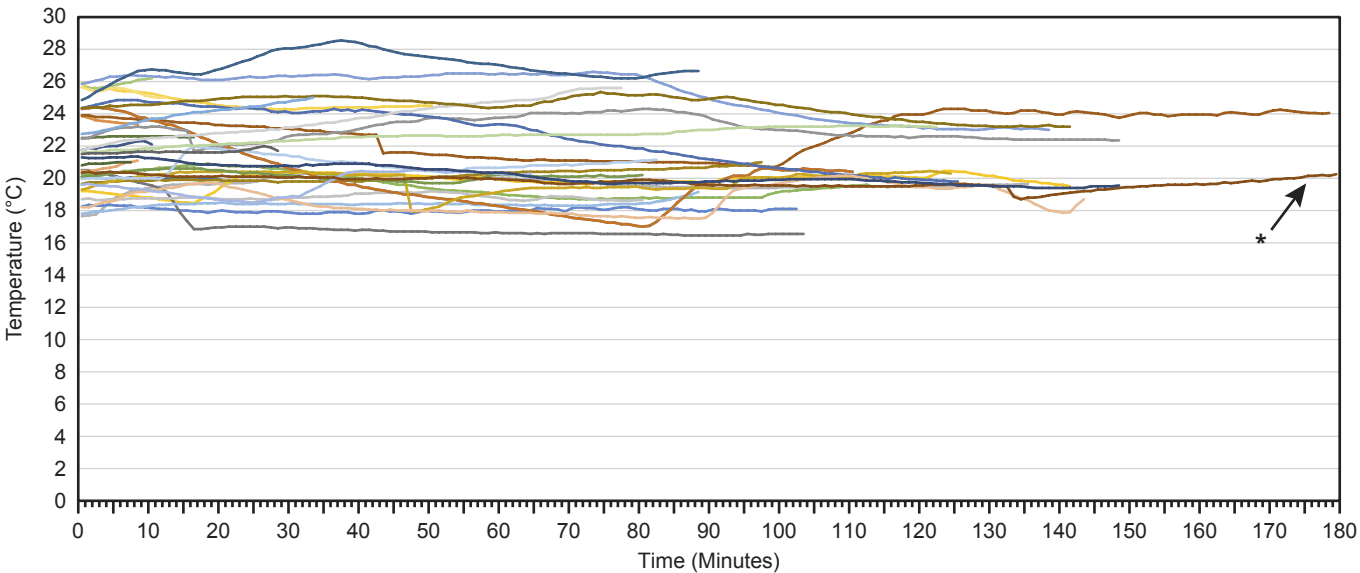

Supplement: Supplementary file 1 — Additional file 1. Additional material showing the temperature plots for all three ambulance vehicles and all four seasons, is available as Additional file 1. The figures are A: HEMS winter, B: HEMS spring, C: HEMS summer, D: HEMS: autumn, E: GA1 winter, F: GA1 spring, G: GA1 summer, H: GA1 autumn, I: GA2 winter, J: GA2 spring, K: GA2 summer, L: GA2 autumn. The asterisk in Fig. G denotes that the graph was truncated at 180 min. The temperature remained essentially unchanged until the end of the mission at 256 min. HEMS: helicopter emergency medical service. GA: ground ambulance. [file 13049_2020_759_MOESM1_ESM.zip › 7-G v2.pdf]

H

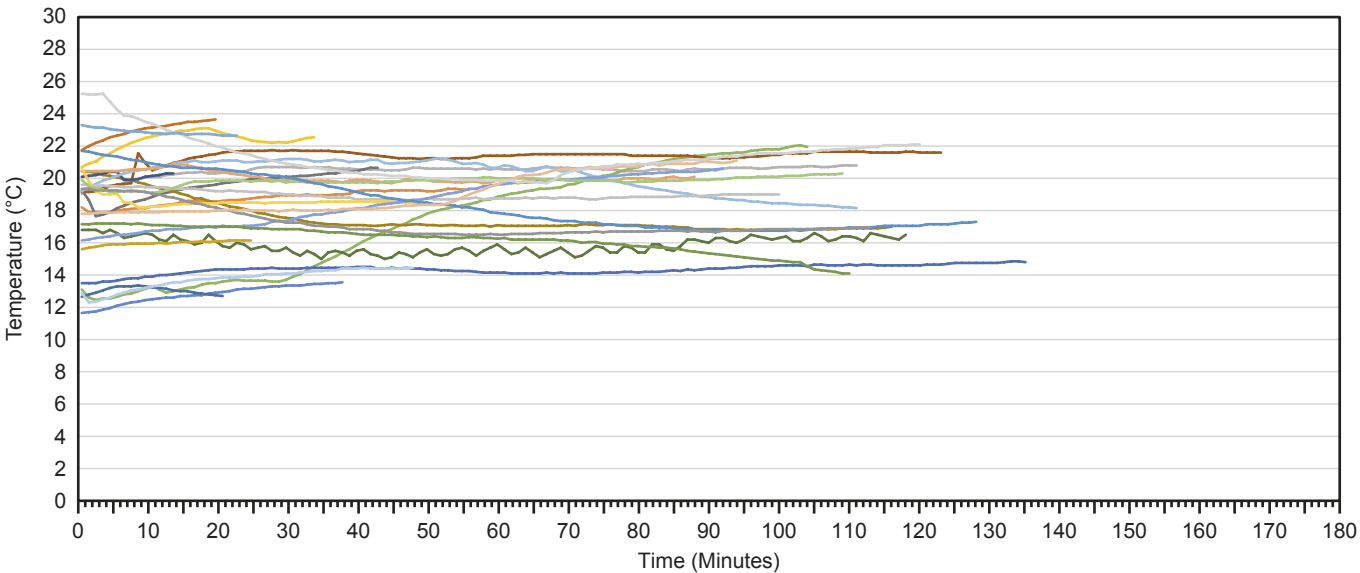

Supplement: Supplementary file 1 — Additional file 1. Additional material showing the temperature plots for all three ambulance vehicles and all four seasons, is available as Additional file 1. The figures are A: HEMS winter, B: HEMS spring, C: HEMS summer, D: HEMS: autumn, E: GA1 winter, F: GA1 spring, G: GA1 summer, H: GA1 autumn, I: GA2 winter, J: GA2 spring, K: GA2 summer, L: GA2 autumn. The asterisk in Fig. G denotes that the graph was truncated at 180 min. The temperature remained essentially unchanged until the end of the mission at 256 min. HEMS: helicopter emergency medical service. GA: ground ambulance. [file 13049_2020_759_MOESM1_ESM.zip › 8-H v2.pdf]

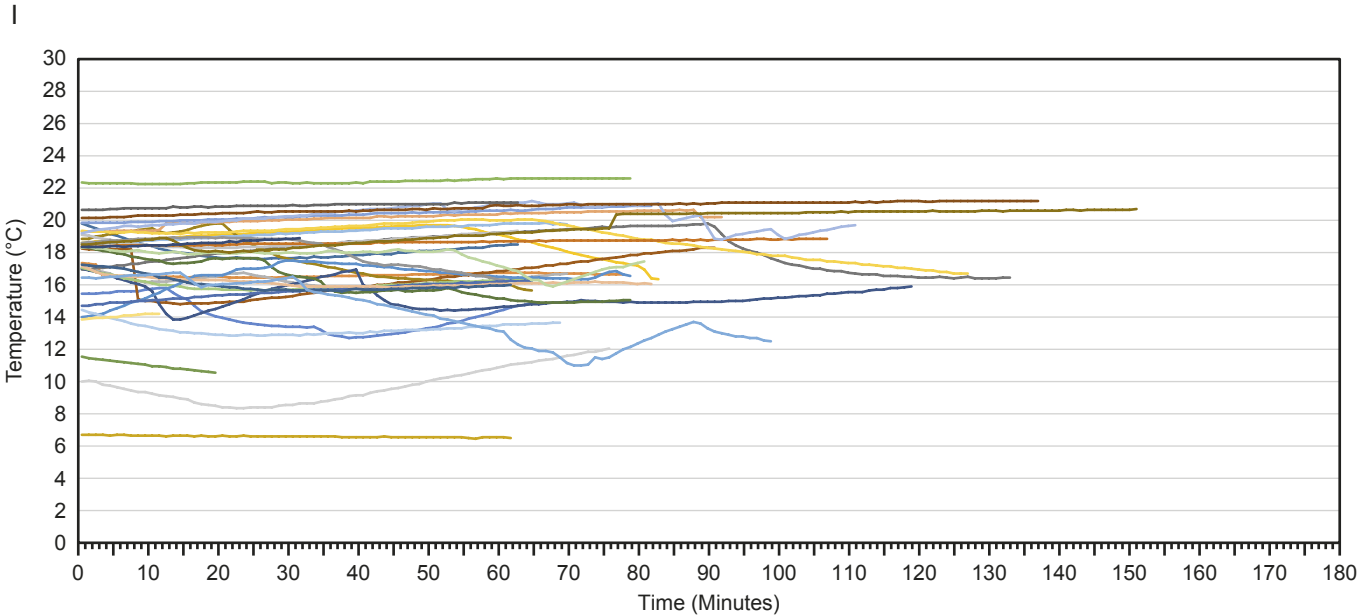

Supplement: Supplementary file 1 — Additional file 1. Additional material showing the temperature plots for all three ambulance vehicles and all four seasons, is available as Additional file 1. The figures are A: HEMS winter, B: HEMS spring, C: HEMS summer, D: HEMS: autumn, E: GA1 winter, F: GA1 spring, G: GA1 summer, H: GA1 autumn, I: GA2 winter, J: GA2 spring, K: GA2 summer, L: GA2 autumn. The asterisk in Fig. G denotes that the graph was truncated at 180 min. The temperature remained essentially unchanged until the end of the mission at 256 min. HEMS: helicopter emergency medical service. GA: ground ambulance. [file 13049_2020_759_MOESM1_ESM.zip › 9-I v2.pdf]
